# Rye-Based Evening Meals Favorably Affected Glucose Regulation and Appetite Variables at the Following Breakfast; A Randomized Controlled Study in Healthy Subjects

**DOI:** 10.1371/journal.pone.0151985

**Published:** 2016-03-18

**Authors:** Jonna C. Sandberg, Inger M. E. Björck, Anne C. Nilsson

**Affiliations:** Food for Health Science Centre, Lund University, SE-221 00, Lund, Sweden; National Institute of Agronomic Research, FRANCE

## Abstract

**Background:**

Whole grain has shown potential to prevent obesity, cardiovascular disease and type 2 diabetes. Possible mechanism could be related to colonic fermentation of specific indigestible carbohydrates, i.e. dietary fiber (DF). The aim of this study was to investigate effects on cardiometabolic risk factors and appetite regulation the next day when ingesting rye kernel bread rich in DF as an evening meal.

**Method:**

Whole grain rye kernel test bread (RKB) or a white wheat flour based bread (reference product, WWB) was provided as late evening meals to healthy young adults in a randomized cross-over design. The test products RKB and WWB were provided in two priming settings: as a single evening meal or as three consecutive evening meals prior to the experimental days. Test variables were measured in the morning, 10.5–13.5 hours after ingestion of RKB or WWB. The postprandial phase was analyzed for measures of glucose metabolism, inflammatory markers, appetite regulating hormones and short chain fatty acids (SCFA) in blood, hydrogen excretion in breath and subjective appetite ratings.

**Results:**

With the exception of serum CRP, no significant differences in test variables were observed depending on length of priming (P>0.05). The RKB evening meal increased plasma concentrations of PYY (0–120 min, P<0.001), GLP-1 (0–90 min, P<0.05) and fasting SCFA (acetate and butyrate, P<0.05, propionate, P = 0.05), compared to WWB. Moreover, RKB decreased blood glucose (0–120 min, P = 0.001), serum insulin response (0–120 min, P<0.05) and fasting FFA concentrations (P<0.05). Additionally, RKB improved subjective appetite ratings during the whole experimental period (P<0.05), and increased breath hydrogen excretion (P<0.001), indicating increased colonic fermentation activity.

**Conclusion:**

The results indicate that RKB evening meal has an anti-diabetic potential and that the increased release of satiety hormones and improvements of appetite sensation could be beneficial in preventing obesity. These effects could possibly be mediated through colonic fermentation.

**Trial Registration:**

ClinicalTrials.gov NCT02093481

## Introduction

Obesity is a major risk factor for developing cardiovascular disease and type 2 diabetes (T2D), and the prevalence of obesity in the world has more than doubled between 1980 and 2014 [1]. Prevention is urgently needed and promising results have been shown with whole grain diets in this context. Epidemiological studies have shown that whole grain intake is associated with reduced risk of cardiovascular disease [2], T2D [3] and lower body mass index (BMI) [4]. One contributing explanation to the health benefits observed with whole grain is connected to the indigestible carbohydrates, i.e. dietary fiber (DF) components, including non-starch polysaccharides (NSP) and resistant starch (RS), e.g. RS1 from intact botanical structure. The DF are fermented by the gut microbiota and among the main metabolites formed are short-chain fatty acids (SCFA), mainly acetate, propionate and butyrate, and gases such as hydrogen [5].

It has been shown that certain whole grain cereal foods rich in DF, e.g. barley kernel products, have the potential to improve important measures of cardiometabolic risk and beneficially affect appetite regulatory hormones in a semi-acute perspective, i.e. 10.5–16 h after consumption, compared to white wheat flour based bread (WWB) [6]. Further, it has been reported that elevated plasma propionate [7], breath hydrogen (H_2_) excretion [8] and plasma levels of butyrate [9], respectively, were positively related to benefits on blood glucose regulation between 10.5–13.5 h [7] and 9.5–11.5 h [8, 9], after ingesting barley kernel meal, compared to WWB. These results points towards a relationship between gut fermentation of DF and metabolic benefits.

Rye-based products have previously shown potential to improve acute postprandial appetite ratings (wholegrain flour- and bran based) [10] and sustained appetite ratings after a 3 week intervention with rye porridge [11] in healthy subjects. In addition, acute postprandial studies in healthy subjects have shown that rye products appear to lower the glucose and insulin response [12, 13] and decrease voluntary food intake at a subsequent meal, i.e. 4.5 h after the rye-based breakfast [14] compared to WWB. The semi-acute effects of rye on blood glucose and breath H_2_ concentration have previously been investigated in a day-long intervention. The results showed that a rye kernel breakfast lowered the blood glucose increment during an 11.5 hour long test day when measuring the cumulative postprandial incremental area under the curve (iAUC) 0–120 min after breakfast, lunch and dinner, compared to after a WWB breakfast. In addition, the rye kernel breakfast increased the breath H_2_ excretion compared to WWB in the same time perspective, indicative of increased gut fermentation [15]. Additionally, an increase in breath H_2_ excretion the following morning after a rye-based evening meal has previously been observed [16], indicating potential over-night gut fermentation of DF in rye. Although rye kernels are rich in DF, e.g. arabinoxylans, fructans, β-glucans and RS, and despite the fact that rye is one of the most commonly consumed cereal in Scandinavian countries [17], few studies have considered the metabolic effect of rye in a semi-acute perspective.

The aim of this study was to investigate potential beneficial effects of colonic fermentation of DF in rye, ingested in the evening, on cardiometabolic risk factors and appetite regulation at fasting and in the postprandial period following a standardized breakfast the next day. For this purpose, a rye kernel based test bread (RKB) or a WWB (reference product) was provided to healthy young adults as evening meals in a randomized cross-over design. Since rye has not previously been studied in this time perspective with respect to effects on cardiometabolic risk factors, two different study settings, which variated in length of priming, were applied to provide the test products. The first setting was an over-night study design, where RKB was provided as a single evening meal before an experimental day the next morning. The second setting was a prolonged intervention where RKB was provided as three consecutive evening meals prior to an experimental day. The prolonged short-term intervention setting was included to investigate if effects on metabolic variables may vary depending on different length of short-term interventions. Both of the study settings mentioned has previously been applied, separately, in studies evaluating cardiometabolic effects of barley kernel based products [6, 8, 18, 19]. In the present study, the test variables were measured in the next morning at the interval of 10.5–13.5 hours after ingestion of the test products. Blood samples were collected at fasting and postprandially, after a subsequent standardized breakfast (experimental day) and were analyzed for measures of test variables involved in e.g. glucose metabolism, appetite regulation, inflammatory tonus and colonic fermentation of DF (plasma SCFA). Additionally, breath H_2_ excretion was measured as a marker of colonic fermentation, and subjective appetite ratings were registered.

## Materials and Methods

### Ethical statement

The study was registered at ClinicalTrials.gov (NCT02093481). Approval of the study was given by the Regional Ethical Review Board in Lund, Sweden (Reference 2013/241). The trial protocol for this study and supporting CONSORT checklist are available as supporting information; see S1 Protocol and S1 CONSORT Checklist.

### Subjects

**Fig 1:**
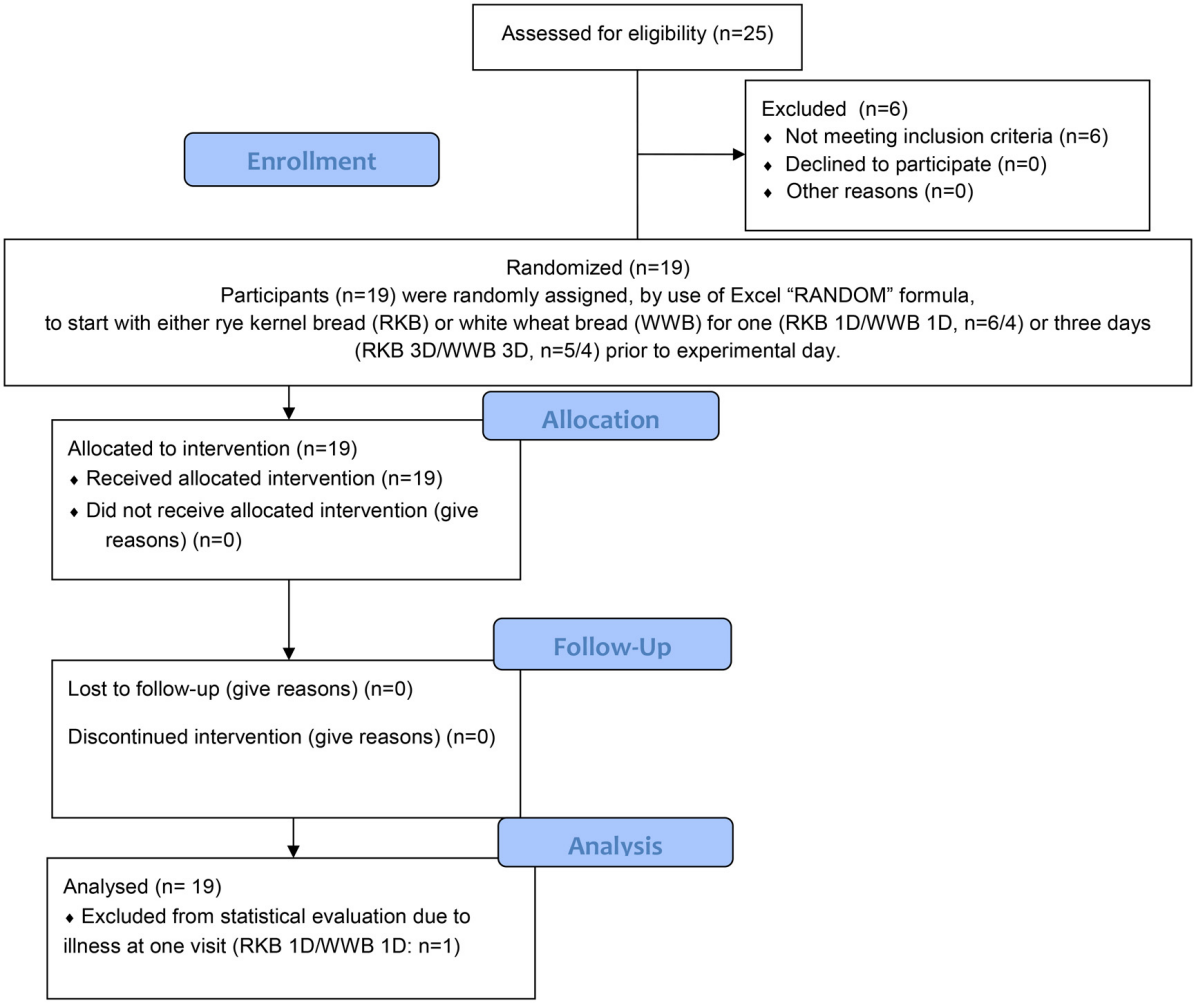
Flow diagram of the study progress.

Nineteen healthy volunteers, 9 men and 10 women aged (mean ± standard deviation (SD)) 25.6 ± 3.5 years, with normal body mass indices (BMI) (mean ± SD: 21.9 ± 1.87 kg/m^2^) were enrolled to participate in the study. Prior to enrollment, six subjects were excluded since one or more of the inclusion criteria were not met. The inclusion criteria were age between 20–35 years, BMI between 19–25 kg/m^2^, non-smoker and no known metabolic disorders or food allergies. Recruitments of test subjects were ongoing between March 10^th^ to March 31^st^ 2014, and the experimental work was finished by June 25^th^ 2014. A flow diagram of the study process is presented in Fig 1. Prior to inclusion, each subject got a full explanation (written and oral) of the purpose and procedure of the study, and written informed consent was obtained from each subject. All test subjects were aware of the possibility of withdrawing from the study at any time they desired.

### Rye kernel test product and WWB reference product

The test product RKB was composed of 85% whole rye kernels and 15% white wheat flour, based on cereal dry matter. A refined wheat bread, WWB, consisting of 100% white wheat flour (based on cereal dry matter) was included as a reference product. The size of the test portions, to be consumed in the evening, was based on 50 g available starch. The rye kernels (commercial blend) were provided by Lantmännen Cerealia (Järna, Sweden), and commercial white wheat flour was obtained from Kungsören AB (Järna, Sweden).

#### RKB

Rye kernels, 654.5 g, were boiled in 520 g water for 30 minutes and then set to cool in room temperature for 30 minutes. All water was absorbed into the kernels when cooked. 115.5 g white wheat flour, 100 g water, 6.6 g yeast (Jästbolaget AB, Sollentuna, Sweden) and 5.5 g NaCl were added to the kernels and were mixed for 4 minutes in a food processor (Electrolux Assistent AKM3110W). The dough was proofed at room temperature for 15 minutes in the dough-mixing bowl and then a second proofing was made in a bread pan for 15 minutes in room temperature. Baking was performed in a house-hold oven at 225°C with steam the first 20 minutes and then the bread was covered with aluminum foil the last 40 minutes (total baking time 60 minutes). The bread was cooled for 2 hours, then sliced into portions (142.5 g bread), wrapped in aluminum foil, put into plastic bags and stored in a freezer (-20°C).

#### WWB

The WWB was baked according to a standardized procedure in a home baking machine (Severin model nr. BM 3983; Menu choice, program 2 [white bread, 1000 g, quick (time2:35)]). The bread was made from 540 g white wheat flour, 360 g water, 4.8 g dry yeast and 4.8 g NaCl. After cooling, the crust was removed and the bread was sliced and portions (121.4 g bread) were wrapped in aluminum foil, put into plastic bags and stored in a freezer (-20°C).

The test subjects stored the bread products in their freezer. At the day of consumption, they were instructed to take a portion from the freezer in the morning and thaw it at ambient temperature, still wrapped in aluminum foil and in the plastic bag. Only water (optional amounts) was allowed to be consumed with the test- and reference products.

### Standardized breakfast

The standardized breakfast consisted of 121.4 g WWB, corresponding to 50 g available starch, and 200 ml of water. The breakfast bread was baked according the same standardized procedure as the reference meal WWB.

### Chemical analyses of evening meals and standardized breakfast

The test products and breakfast were analyzed with respect to *total starch* according to Björck et al. (1992) [20], *RS* according to Åkerberg et al. (1998), which included chewing as a pre-step before incubation with enzymes [21], and *NSP* (soluble and insoluble) analyzed with a gravimetric, enzymatic method according to Asp et al. (1983) [22]. Information regarding the composition of the test and reference meals is provided in Table 1. Before analysis of total starch and DF, the bread products were air dried at room temperature for 16 hours and milled (Cyclotec, Foss Tecator AB, Höganäs, Sweden). RS in all test products was analyzed on products as eaten. The available starch content was calculated by subtracting RS from the total starch.

**Table 1:** Carbohydrate composition and portion size of the test- and reference products respectively^1^.

Product	Portion size	Starch			NSP		Total DF
		Total	Available	RS	Insoluble	Soluble	
**——————————% *dry matter*——————————**							
RKB	-	66.6	61.8	4.80	12.3	3.81	21.0
WWB	-	80.5	78.5	2.02	2.57	2.00	6.59
**————————————*g/day*————————————**							
RKB	142.5	53.9	50.0	3.89	8.98	2.77	15.6
WWB	121.4	51.3	50.0	1.29	1.47	1.14	3.89

^1^ Data are presented as means.

Available starch is calculated as the difference between total starch [20] and RS [21]. Values of total starch are based on means of 2 replicates, RS means of 6 replicates and DF are based on means of 2 replicates. Insoluble and soluble DF was determined gravimetrically according to Asp *et al*. (1983) [22]. Total DF include RS, and insoluble and soluble NSP. RKB, rye kernel based bread; DF, dietary fiber; NSP, non-starch polysaccharides; RS, resistant starch; WWB, white wheat flour based reference bread.

### Study design and procedure

The design was randomized cross-over, with approximately 1 week in-between two test occasions. The RKB and WWB were consumed as evening meals at 0930 pm. Thereafter the test subjects were fasting until the breakfast was served the following morning at the research unit at the Food for Health Science Centre (former division of Applied Nutrition and Food Chemistry), Lund University. The test subject consumed each product twice, applying two different priming settings prior to the experimental day; i) test- and reference products provided as single evening meals, ii) test- and reference products provided as evening meals during 3 consecutive days. To separate the two different approaches of consumption, the evening meals ingested as a single evening meal prior to experimental day were abbreviated RKB 1D (RKB one day) and WWB 1D, and the evening meals ingested during 3 consecutive evenings prior to the experimental day were denoted RKB 3D (RKB three days) and WWB 3D. In total, the test subjects participated at four occasions. Test subjects were randomly assigned the evening meals and length of priming, by use of Excel’s *Random* formula. The number of test subjects assigned to start with each test product was divided accordingly: RKB 1D (6 subjects), WWB 1D (4 subjects), RKB 3D (5 subjects) or WWB 3D (4 subjects).

During the experimental period, the subjects were encouraged to standardize their meal pattern and to maintain their regular eating habits. To simplify the standardization of meal patterns, test subjects were asked to provide meal records during three days prior to the experimental day. They were also instructed to avoid alcohol and food rich in DF three days prior to experimental day, and to avoid excessive physical exercise the day prior to the experimental day. No antibiotics or probiotics were allowed within two weeks before or during the study period (no test subject had consumed antibiotics during the last month). At the experimental days, the subjects arrived to the research unit at 0730 am. An intravenous cannula (BD Venflon^™^ Pro Safety Shielded IV Catheter, Becton Dickinson) was inserted into an antecubital vein for blood sampling. Fasting blood samples were collected and appetite and breath H_2_ excretion were registered before the breakfast. A standardized breakfast was served at 0800 am and finished within 15 min. The test subjects were told to sustain a low physical activity during the 3 hours of repeated sampling of test variables.

### Physiological test parameters

Fasting and postprandial measurements were performed at all four visits. Finger-prick capillary blood samplings were applied for determination of blood glucose (HemoCue^®^B-glucose, HemoCue AB, Ängelholm, Sweden). Venous blood samples were collected to determine serum (s-) insulin, s-FFA, s-CRP, s-IL-6, s-IL-18, s-adiponectin, s-PAI-1, s-TG, plasma (p-) GLP-1 (active 7–36), p-PYY, p-ghrelin, p-GLP-2, p-nesfatin-1 and p-SCFA. Venous blood samples intended for analysis of GLP-1, PYY and GLP-2 were collected in tubes with added DDPIV-inhibitor (10 μl/ml blood) (Millipore, St Charles, USA) and the separated plasma was stored at -80°C. Samples intended for analysis of ghrelin were collected together with aprotinin (50 μl/ml blood) (Sigma-Aldrich, St Louis, USA) and the plasma was treated with 1 M HCl (10:1) prior to freezing (-80°C). The remaining serum- and plasma samples were stored at -40°C prior to analysis. Breath hydrogen was measured as an indicator of colonic fermentation, using an EC 60 gastrolyzer (Bedfont EC60 Gastrolyzer, Rochester, England). Pre- and post-breakfast subjective appetite sensations (satiety, hunger and desire to eat) were obtained using a 100 mm Visual Analogue Scale (VAS). Blood glucose, breath H_2_, VAS ratings for subjective appetite (hunger, satiety and desire to eat) were obtained at fasting (time 0 min) and at 15, 30, 45, 60, 90, 120 and 180 minutes after commencing the breakfast, and s-insulin was determined on similar time points, excluding 15 min. Plasma ghrelin and s-triglycerides were determined at time 0 min and at 30, 60, 120 and 180 min after breakfast. Plasma GLP-1 and p-GLP-2 were determined at time 0 min and every half hour until 120 min. Serum adiponectin was obtained at time 0 min and every hour until 180 min, and p-PYY was determined at time 0 min and every hour until 120 min post breakfast. Serum IL-6 and s-IL-18 were determined at time 0 min and at 120 and 180 min, and s-PAI-1, s-FFA and s-CRP were obtained at time 0 min and at 180 min. Plasma SCFA and p-nesfatin-1 were determined at time 0 min.

*Serum insulin* was determined with a solid phase two-site enzyme immunoassay kit (Insulin ELISA 10-1113-01, Mercodia AB, Uppsala, Sweden) and *s-FFA* concentrations with an enzymatic colorimetric method using a 96 well microplate (NEFA C, ACS-ACOD method, WAKO Chemicals GMbH, Germany). The quantitative determination of *s-IL-6* was performed with an enzyme immunoassay (Human IL-6 HS600B, R&D Systems, Abingdon, UK) and *IL-18* in serum with an enzyme immunoassay that was modified in the sense that no dilution of serum was performed prior to the analysis (Human IL-18 ELISA Kit 7620, MBL Medical & Biological Laboratories CO., Ltd, Nagoya, Japan). Enzyme immunoassay was also used to determine *PAI-1* in serum (Human PAI-1 ELISA, RBMS2033R, BioVendor GmbH, Heidelberg, Germany) and *s-CRP* (CRP ELISA Kit, Immunodiagnostik AG, Bensheim, Germany). *Serum Triglycerides* were analyzed with a multi-sample enzymatic assay (LabAssay^™^ Triglyceride 290–63701, GPO·DAOS method, Wako Chemicals GmbH, Neuss, Germany) and *serum adiponectin* concentrations were determined with a solid phase two-site enzyme immunoassay kit (Adiponectin ELISA 10-1193-01, Mercodia AB, Uppsala, Sweden). *Plasma GLP-2* concentrations were determined with a competitive enzyme immunoassay (Human GLP-2 EIA YK141, Yanaihara Institute Inc. Shizuoka, Japan) and *p-ghrelin* with an enzyme immunometric assay (Human Acylated Ghrelin ELISA RD194062400R, BioVendor GmbH, Heidelberg, Germany). The quantitative determination of bioactive *GLP-1* levels in plasma was made with a highly sensitive ELISA enzyme-linked immunosorbent assay kit (GLP-1 (Active 7–36) ELISA 43-GP1HU-EO1 ALPCO Diagnostics, Salem, NH). The concentrations of *PYY*, both PYY (3–36) and PYY (1–36), in plasma were determined with a competitive enzyme immunoassay (Human PYY EIA YKO8O, Yanaihara Institute Inc. Shizuoka, Japan) and *nesfatin-1* in plasma with a sandwich enzyme immunoassay (Human Nesfatin-1 ELISA RD191227200R, BioVendor GmbH, Heidelberg, Germany). Plasma SCFA (acetate, propionate and butyrate) were analyzed using a GC method [23].

### Calculations and statistical methods

Data are expressed as means ± SEM. Statistical evaluations involving glucose and insulin concentrations are based on incremental changes from fasting concentrations. The absolute values are used in the statistical calculations of the rest of the test variables. The incremental area- and area under the curve (iAUC and AUC, respectively) were calculated for each subject and test meal, using the trapezoid model. The AUC was used in the statistical evaluation of ghrelin results and parameters for appetite sensations. The iAUC was used in the statistical evaluation of results regarding blood glucose and insulin, since the mentioned test variables are not normally affected significantly at fasting in healthy people. The fasting values were statistically evaluated before using iAUC instead of AUC. For calculation of incremental responses, the fasting value at the day of measurements was used as baseline. Incremental peak (iPeak) concentrations were calculated for glucose and insulin as individual maximum postprandial increase from the baseline. GraphPad Prism (version 6, GraphPad Software, San Diego, CA, USA) was used for graph plotting and calculation of AUC.

Significant differences in test variables depending on the different test meals were assessed with ANOVA (general linear model) and the data was analyzed in MINITAB Statistical Software (release 17; Minitab, Minitab Inc, State College, PA) as a 2x2 factorial design. The test products (RKB and WWB), and length of priming (1D and 3D), were used as independent main variables, and in addition, interactions between these independent variables were observed. Test subjects were included as a random factor in the model. The significance level was set at *P*-values ≤ 0.05. If a significant main effect of test product was detected, and no significant interactions were seen, it will be referred to as a significant main effect of *test product*, and the test- and reference products will be referred to as ‘RKB’ and ‘WWB’, respectively. If a significant main effect of *days* was observed, data will be presented as ‘1D’ including the mean of WWB 1D and RKB 1D and as ‘3D’ including WWB 3D and RKB 3D data. The test products were only distinguished as RKB 1D and WWB 1D, respective RKB 3D and WWB 3D if a significant [length of priming*product] interaction was detected. In the cases of unevenly distributed residuals (tested with Anderson-Darling and considered unevenly distributed when *P*<0.05), a Box Cox transformation was performed on the data prior to the ANOVA analysis. Differences between the products (‘Meal’: RKB and WWB) at different time points during the experimental day (‘Time’) were evaluated using a mixed model (PROC MIXED in SAS release 9.3; SAS Institute Inc, Cary, NC) with repeated measures and an autoregressive covariance structure. The relationships between SCFA (total and individual, i.e. acetate, propionate and butyrate) and relevant gut hormones, i.e. gut hormones with a significant main effect of test product were investigated using Pearson correlation in Minitab statistical software (release 17; Minitab, Minitab Inc, State College, PA). If a result of a test variable from a test subject was missing for one of the test- or reference products, the test subject was excluded from the statistical calculations of that specific test variable and length of priming. For example, if a subject was missing a value from a test variable connected to test product WWB 1D, that subject was excluded from both WWB 1D and RKB 1D.

The insulin sensitivity was obtained by calculating the composite insulin sensitivity index (ISI_composite_), so called Matsuda index, using the formula: 10 000/square root of [fasting glucose (mg/dl) × fasting insulin (μU/ml) × mean glucose concentrations 0–120 min (mg·min/dl) × mean insulin concentrations 0–120 min (μU·min/ml)] [24]. To obtain mean glucose- and insulin concentrations in the formula, blood glucose and s-insulin were measured every 30 minutes in the postprandial phase after the standardized breakfast. Thus, the determination of ISI_composite_ was modified by ingesting WWB corresponding to 50 g available carbohydrates for breakfast instead of 75 g of glucose. For breath H_2_, where the variation in the concentration changed scarcely over time, a weighted mean was produced by calculating a mean for equal time intervals (1 mean per hour) over the test period, and then a mean for the different hours was calculated and used in the statistical analysis.

One subject was excluded from the statistical analysis including single evening meals (1D) (WWB 1D and RKB 1D, n_1D_ = 18). This was because it was discovered that after the completed experimental period the subject had an upper respiratory tract infection during one visit which probably may have affected the results. Additionally, due to technical difficulties when analyzing data for GLP-1, time points 60 and 120 min were excluded, and AUC will not be presented in the statistical evaluation but instead as an overall mean for fasting and the postprandial phase 30 and 90 minutes. One subject was removed from statistical analysis of GLP-1 in the 3D test occasions due to an analytical error. In the analysis of nesfatin-1, some subjects were excluded due to values being outside the detection level of the analysis protocol, resulting in n_1D_ = 15 and n_3D_ = 17. Due to technical difficulties with the blood glucose reading machine at one test occasion and analytical problem with insulin determinations, the statistical analysis of glucose and insulin, respectively, included 17 subjects for 1D test situations and 18 subjects for 3D occasions. Two subjects were excluded from statistical analysis of subjective ratings of appetite sensations due to misconception of the scale at the first visit and a missing value at two visits, respectively, (n_1D_ = 17 and n_3D_ = 18).

### Power Calculation

Primary outcome measure was change in blood glucose, iAUC, 0–120 min after the standardized breakfast. The number of test subjects required for the study was determined in MINITAB, using previous results from a study including evening test meals consisting of barley kernels and the effects the following morning on glucose (iAUC 0–120 min) [8]. Assuming a difference of 70 mmol*min/L between after consuming WWB or RKB the previous evening, and a SD of 82 mmol*min/L, with α = 0.05 and 1−β = 0.8, a number of 13–17 test subjects were required (two-tailed test). We decided to increase the number of test subjects to 19 due to the scarce investigations of semi-acute effects on metabolic variables of rye.

## Results

### Blood glucose, serum insulin and insulin sensitivity (ISI_composite_)

**Fig 2:**
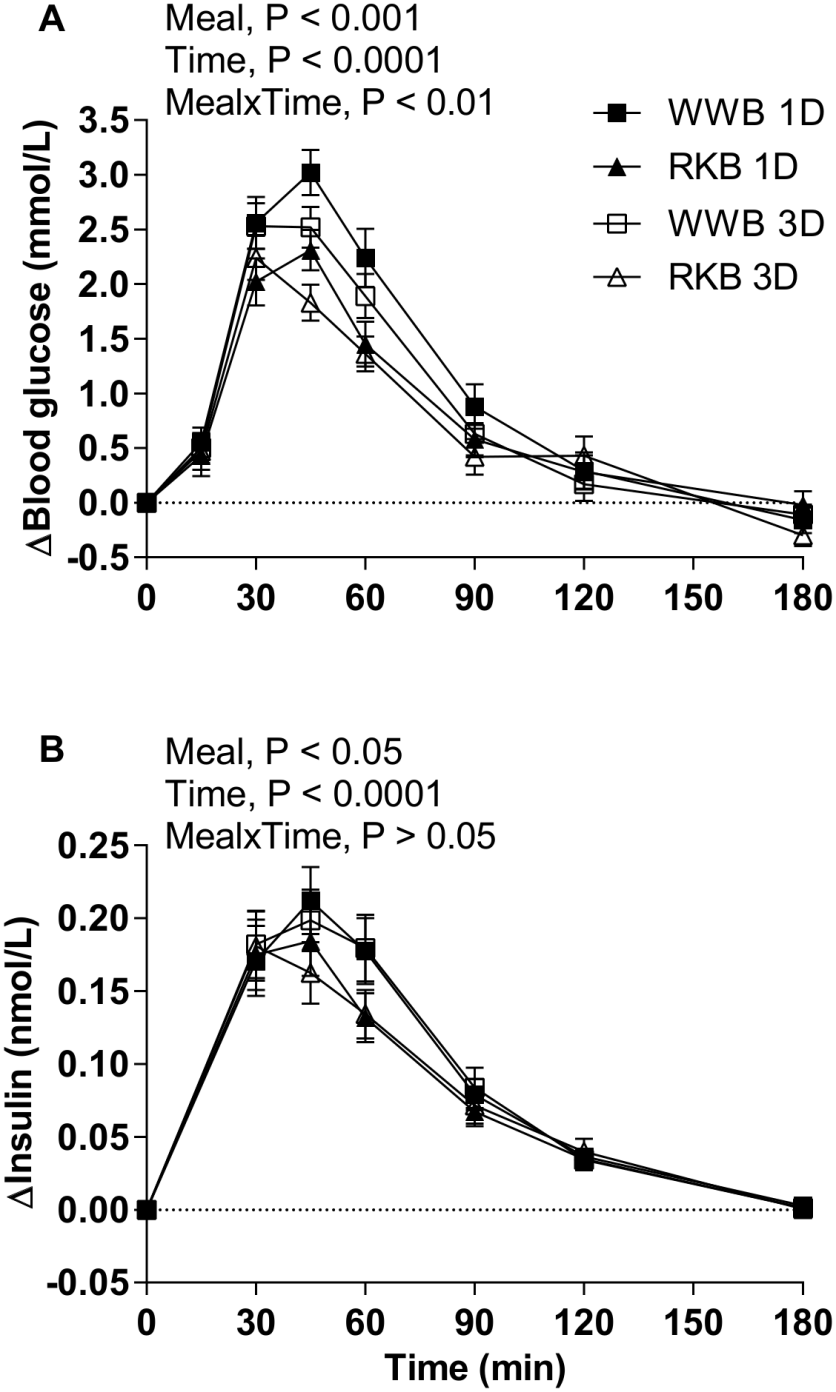
Incremental blood glucose—and serum insulin response after the standardized breakfast. Mean incremental blood glucose (**A**) and serum insulin (**B**) changes (Δ) from fasting concentrations the next morning after one (1D) or three consecutive evening meals (3D) with RKB or WWB, respectively. Values are means ± SEM. Repeated measures; mixed model in SAS. RKB, rye kernel bread; WWB, white wheat bread.

Fig 2 and Table 2 displays that there was a main effect of evening meal test products on blood glucose iAUC (0–120 min, P = 0.001) and the blood glucose iPeak (P<0.01), showing lower postprandial blood glucose responses at the standardized breakfast when preceded by an evening meal with RKB compared to WWB. In addition, in comparison to the WWB evening meal, the RKB evening meal reduced s-insulin iAUC 0–120 min after the standardized breakfast (P<0.05). No significant increase in insulin sensitivity, as obtained by Matsuda index, was observed at breakfast, after the RKB evening meal compared to WWB (Table 2). No differences in concentration were observed between fasting blood glucose or fasting s-insulin depending on previous evening meal (Table 2). No main effects of length of priming (1D or 3D), or [length of priming*product] interactions, were observed in neither blood glucose nor s-insulin values.

**Table 2:** Blood glucose and s-insulin concentrations including insulin sensitivity following one and three consecutive days of evening meals with reference- and test product, respectively^1^.

Test variables	WWB	RKB	%Δ^2^
Fasting blood glucose, (mmol/L)^3^	5.1±0.1	5.2±0.1	2
Blood Glucose, iAUC 0–120 min (mmol·min/L) ^3^	163±11	125±7	−23**
Blood Glucose, iPeak (mmol/L) ^3^	3.1±0.1	2.6±0.1	−16**
Fasting s-insulin (nmol/L) ^3^	0.032±0.002	0.031±0.002	−3
s-Insulin, iAUC 0–120 min (nmol·min/L) ^3^	14.0±0.9	12.2±0.8	−13*
s-Insulin, iPeak (nmol/L) ^3^	0.24±0.02	0.22±0.02	−8
Insulin Sensitivity Index (ISI_composite_) ^3^	10.2±0.5	11.2±0.7	10

^1^ Data are presented as means ± SEM.

* Different from WWB P<0.05;

** P<0.01 (ANOVA, GLM, Minitab).

^2^ The percentage change is calculated as the difference from the WWB to the RKB.

^3^ n_1D_ = 17, n_3D_ = 18. b, whole blood; s, serum; RKB, rye kernel based bread including one and three days of priming; iAUC, incremental area under curve; iPeak, incremental peak; WWB, white wheat bread including one and three days of priming.

### Gastrointestinal hormones and nesfatin-1

**Fig 3:**
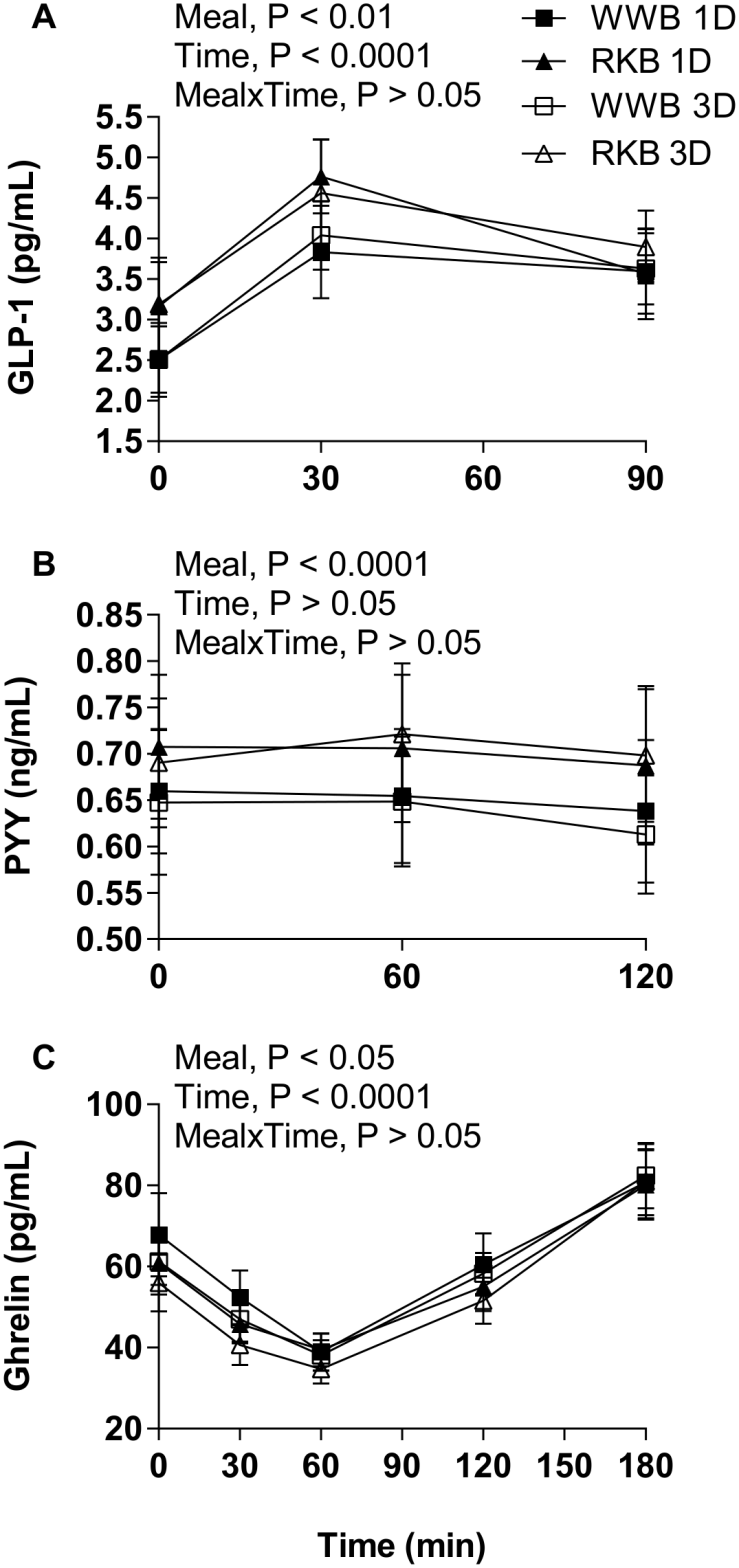
p-GLP-1, p-PYY and p-ghrelin response after breakfast. Mean concentrations of plasma GLP-1 (**A**), p-PYY (**B**) and p-ghrelin (**C**) post ingestion of one or three evening meals consisting of RKB or WWB. Values are means ± SEM. Repeated measures; mixed model in SAS. p, plasma; RKB, rye kernel bread; WWB, white wheat bread; 1D, single evening meal; 3D, three consecutive evening meals.

Regarding gastrointestinal hormones, a significant main effect of test product on p-PYY was observed at fasting (P<0.01) and during the experimental period 0–120 minutes after the standardized breakfast (P<0.001), revealing higher concentrations of p-PYY after RKB evening meals compared with after WWB evening meals (Fig 3 and Table 3). In addition, there was a main effect of test product on p-GLP-1 concentrations at fasting (P<0.05) and 0–90 min after the standardized breakfast (P<0.01), showing significantly increased p-GLP-1 with RKB compared to WWB (Fig 3 and Table 3). The p-ghrelin fasting concentrations did not differ significantly depending on previous evening meal. However, a tendency towards lower postprandial concentrations of p-ghrelin was observed 0–180 min after the breakfast in the case of the RKB evening meal (AUC 0–180 min, P = 0.086), see Fig 3 and Table 3. There was no significant difference at fasting or in the mean 0–120 min response of p-GLP-2 concentrations for the test product (Table 3). A trend towards increased concentrations of p-nesfatin-1 at fasting after a RKB evening meal compared to WWB (P = 0.095) was shown (Table 3). No main effects of length of priming (1D or 3D), or [length of priming*product] interactions, were observed for any of the gastrointestinal hormones analyzed.

**Table 3:** Gastrointestinal hormones and nesfatin-1 responses following one and three consecutive days of evening meals with reference- and test product, respectively^1^.

Test variables	WWB	RKB	%Δ^2^
p-PYY, fasting (ng/mL)^3^	0.65±0.05	0.69±0.05	6**
p-PYY, mean 0–120 min (ng/mL)^4^	0.64±0.05	0.70±0.05	9***
p-GLP-1, fasting (pg/mL)^5^	2.6±0.3	3.2±0.4	23*
p-GLP-1, mean 0–90 min (pg/mL)^5^	3.4±0.3	3.8±0.3	12**
p-Ghrelin, fasting (pg/mL)^3^	64.4±5.7	58.4±5.2	−9
p-Ghrelin, AUC 0–180 min (pg·min/mL)^3^	10200±717	9450±626	−7
p-GLP-2, fasting (ng/mL)^3^	24.3±1.6	24.6±1.5	1
p-GLP-2, mean 0–120 min (ng/mL)^5^	24.6±1.5	24.6±1.4	0
p-Nesfatin-1, fasting (ng/mL)^6^	3.4±0.8	3.5±0.9	3

^1^ Data are presented as means ± SEM.

* Different from WWB P<0.05;

** P<0.01,

*** P<0.001 (ANOVA, GLM, Minitab).

^2^ The percentage change is calculated as the difference from the WWB to the RKB.

^3^ n_1D_ = 18, n_3D_ = 19.

^4^ n_1D_ = 18, n_3D_ = 17.

^5^ n_1D_ = 18, n_3D_ = 18.

^6^ n_1D_ = 15, n_3D_ = 17.

p, plasma; RKB, rye kernel based bread including one and three days of priming; GLP, glucagon-like peptide; PYY, peptide YY; WWB, white wheat bread including one and three days of priming.

### Blood lipids

A tendency was found towards a meal*time interaction for the s-FFA in the postprandial period after the standardized breakfast (P = 0.052, repeated measures; mixed model in SAS), revealing a higher serum FFA in the fasting state when the subjects consumed RKB as an evening meal compared to the WWB (P<0.05, Table 4). There was no significant difference in concentrations of s-FFA and s-TG following the standardized breakfast, or of s-TG at fasting, depending on the evening products (Table 4). There were no significant effects on s-FFA or s-TG depending of the length of priming. No main effects of length of priming (1D or 3D), or [length of priming*product] interactions, were observed with respect to s-FFA and s-TG concentrations.

**Table 4:** Blood lipids, inflammation variables and s-PAI-1 concentrations following one and three consecutive days of evening meals with reference- and test product, respectively^1^.

Test variables	WWB	RKB	%Δ^2^
s-FFA, fasting (mmol/L) ^3^	0.57±0.04	0.49±0.03	−14*
s-FFA, mean 0+180 min (mmol/L) ^3^	0.45±0.03	0.41±0.02	−9
s-Triglycerides, fasting (mg/dL) ^3^	89.5±5.8	88.8±6.9	−1
s-Triglycerides, mean 0–180 min (mg/dL) ^4^	86.3±5.4	88.6±6.7	3
s-Adiponectin, fasting (mg/L)^3^	10.2±0.5	10.2±0.6	0
s-Adiponectin, mean 0–180 min (mg/L) ^3^	10.2±0.5	10.2±0.6	0
s-IL-6, fasting (pg/mL)^4^	0.64±0.06	0.68±0.07	6
s-IL-6, mean 0–180 min (pg/mL)^4^	1.45±0.20	1.18±0.10	−19
s-IL-18, fasting (pg/mL)^3^	187±13	197±15	5
s-IL-18, mean 0–180 min (pg/mL) ^3^	190±13	198±15	4
s-PAI-1, fasting (ng/mL)^4^	329±72	312±52	−5
s-PAI-1, mean 0+180 min (ng/mL)^4^	331±70	303±49	−8
s-CRP, fasting (ng/mL)^5^	464±89	473±95	2
s-CRP, mean 0+180 min (ng/mL)^5^	464±88	455±91	−2

^1^ Data are presented as means ± SEM.

* Different from WWB P<0.05 (ANOVA, GLM, Minitab).

^2^ The percentage change is calculated as the difference from the WWB to the RKB.

^3^ n_1D_ = 18, n_3D_ = 19.

^4^ n_1D_ = 17, n_3D_ = 19.

^5^ n_1D_ = 17, n_3D_ = 18.

s, serum; RKB, rye kernel based bread including one and three days of priming; FFA, free fatty acids; IL, interleukin; PAI-1, plasminogen activator inhibitor; CRP, C-reactive protein; WWB, white wheat bread including one and three days of priming.

### Inflammatory variables and endothelial function

A significant main effect (type of evening meal) was found over the test period (P<0.01, repeated measures; mixed model in SAS) of IL-18. However, there was no significant difference in fasting- or mean serum concentrations of IL-6, IL-18, adiponectin or PAI-1 0–180 min following the standardized breakfast depending on the preceding evening products, or length of priming, see Table 4. Neither did the evening products differ in effect on s-CRP concentrations in the morning (Table 4). However, there was a main effect of length of priming showing that three days of priming (3D) with RKB and WWB significantly decreased the fasting- and mean serum levels of s-CRP 0–180 min post breakfast (P<0.05) compared to one day of priming (1D). The fasting concentrations of s-CRP were 596±112 and 347±62 ng/ml for 1D and 3D designs, respectively, and the mean concentrations (0–180 min post breakfast) were 584±109 ng/ml for 1D compared to 342±61 ng/ml for 3D. No main effect of [length of priming*product] interactions was observed for any of the test variables mentioned above.

### Plasma SCFA

**Fig 4:**
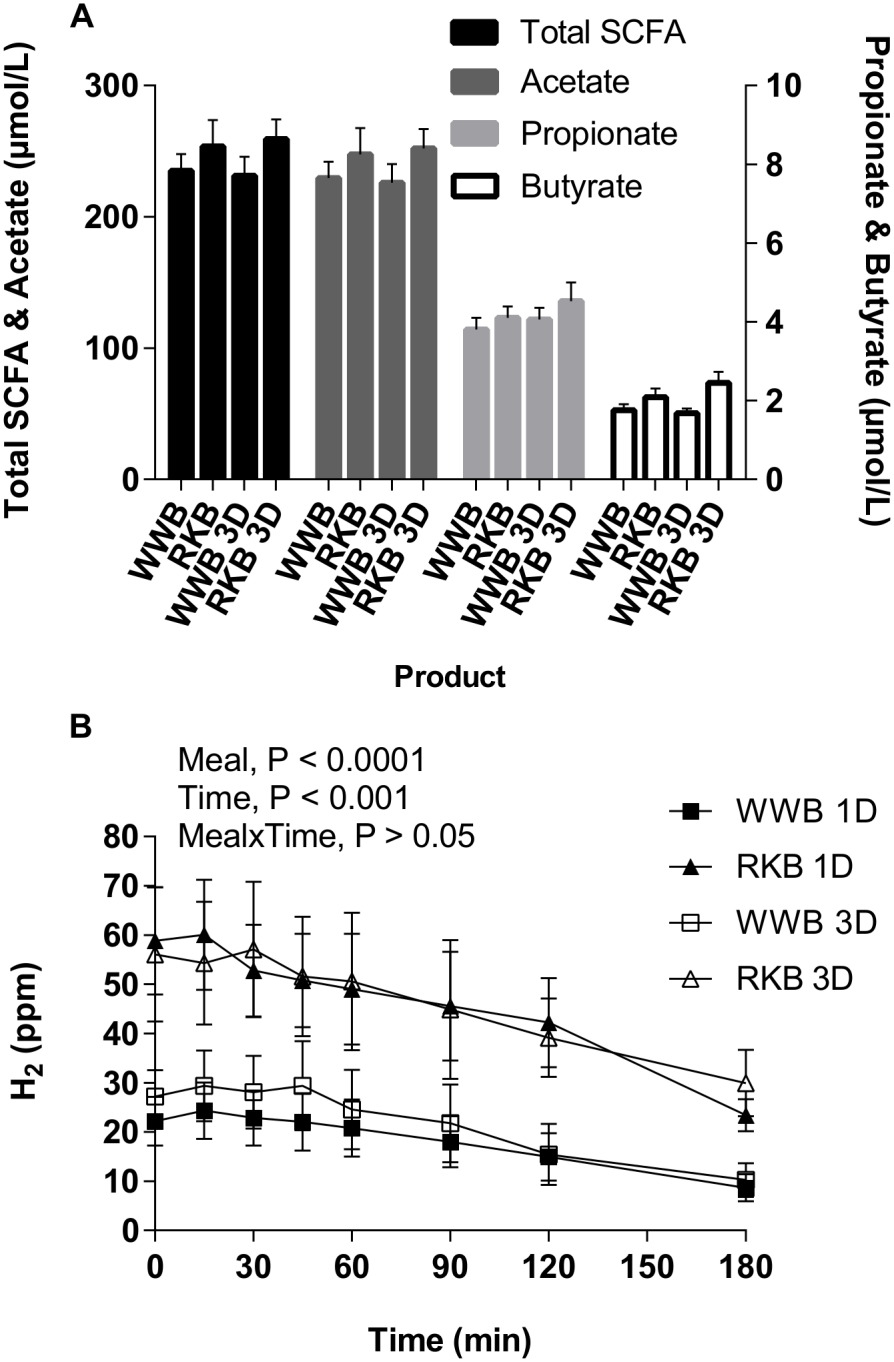
SCFA response at fasting and breath H_2_ response during the experimental day. Fasting concentrations of total SCFA and acetate on the left y-axis, and of propionate and butyrate on the right y-axis (**A**) and mean postprandial breath H_2_ concentrations (**B**) at a standardized breakfast, following the evening meals with RKB or WWB ingested as a single evening meal (1D) or during three consecutive evenings (3D) prior to experimental day. Values are means ± SEM. Repeated measures; mixed model in SAS. p, plasma; RKB, rye kernel bread; SCFA, short-chain fatty acids; WWB, white wheat bread.

At fasting, the p-SCFA were significantly higher after the RKB evening meal compared to WWB (Fig 4 and Table 5). Consequently, the total p-SCFA concentrations (mean of acetate, propionate and butyrate, P<0.05) increased as well as individual p-SCFA; acetate (P<0.05), propionate (P = 0.05), butyrate (P<0.01), see Table 5. No significant differences were seen depending on length of priming. However, a close to significant increase in p-propionate concentrations was observed with 3D priming compared with 1D (P = 0.051, Fig 4). The fasting concentrations of total p-SCFA, acetate and propionate were positively correlated with mean concentration of p-GLP-1 after RKB (total SCFA: r = 0.35, P = 0.036, acetate: r = 0.35, P = 0.039 and propionate: r = 0.36, P = 0.031). No main effect of [length of priming*product] interactions was observed.

**Table 5:** Plasma SCFA and breath H_2_ following one and three consecutive days of evening meals with reference- and test product, respectively^1^.

Test variables	WWB	RKB	%Δ^2^
p-total SCFA, fasting (μmol/L)^3^	234±10	258±13	10*
p-Acetate, fasting (μmol/L)^3^	229±10	251±13	10*
p-Propionate, fasting (μmol/L)^3^	3.99±0.21	4.38±0.28	10^†^
p-Butyrate, fasting (μmol/L)^3^	1.74±0.10	2.27±0.18	30**
breath H_2_, fasting (ppm)^3^	22.5±3.2	58.6±8.3	160***
breath H_2_, mean 0–180 min (ppm)^4^	17.3±3.7	42.1±6.4	143***

^1^ Data are presented as means ± SEM.

* Different from WWB P<0.05;

** P<0.01,

*** P<0.001,

^†^ P = 0.05 (ANOVA, GLM, Minitab).

^2^ The percentage change is calculated as the difference from the WWB to the RKB.

^3^ n_1D_ = 18, n_3D_ = 19.

^4^ n_1D_ = 17, n_3D_ = 16.

p, plasma; RKB, rye kernel based bread including one and three days of priming; SCFA, short chain fatty acids; H_2_, hydrogen gas; WWB, white wheat bread including one and three days of priming.

### Breath H_2_ excretion

The mean H_2_ excretion was significantly higher at fasting and in the postprandial period after breakfast (0–180 min) following an RKB evening meal compared with WWB (P<0.001) with no significant difference depending on the length of priming (Table 5 and Fig 4). No main effect of [length of priming*product] interactions was observed.

### Subjective ratings of satiety, hunger and desire to eat

**Fig 5:**
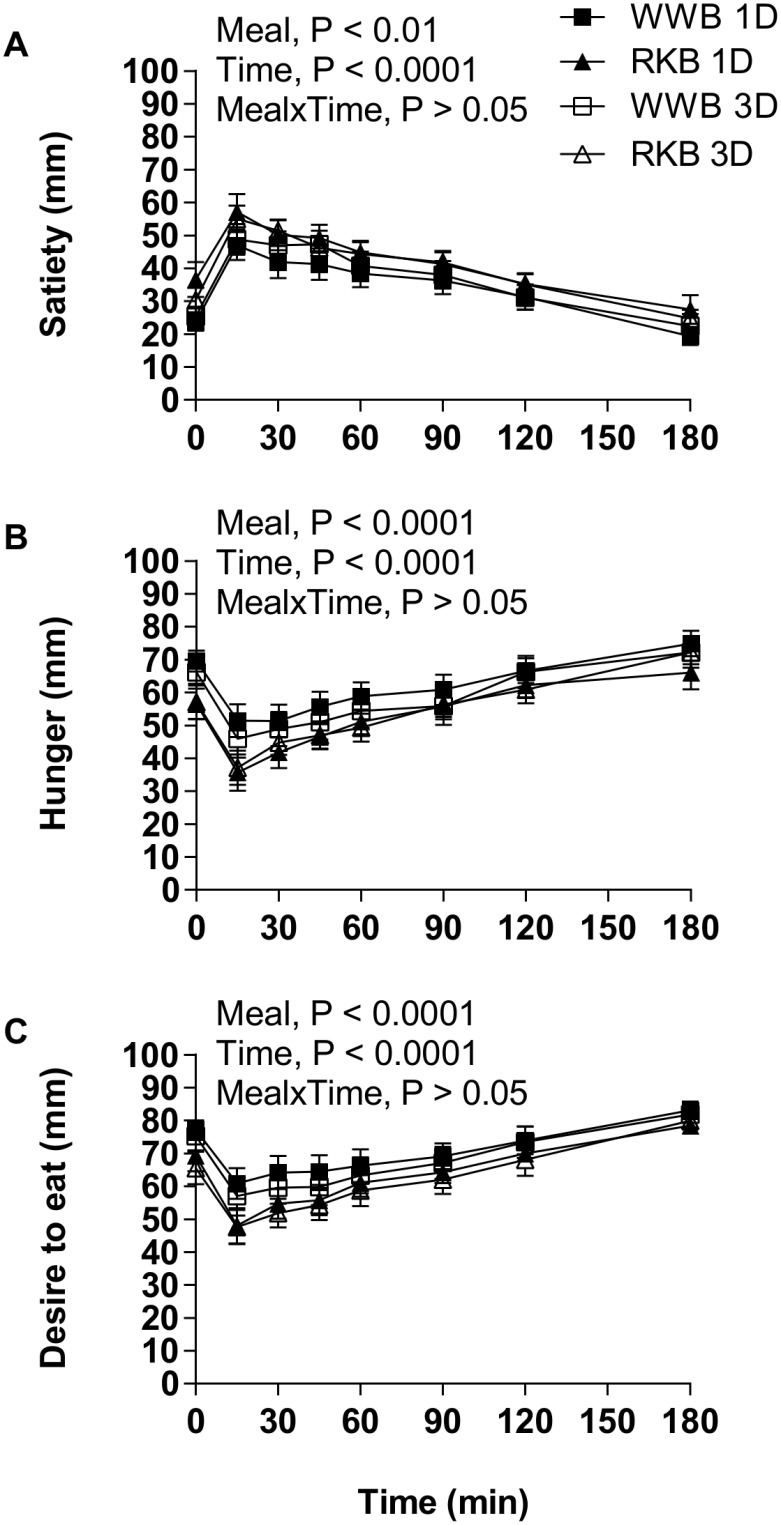
Postprandial responses of satiety, hunger and desire to eat during the experimental day. Mean of subjective appetite ratings (VAS) of satiety (**A**), hunger (**B**) and desire to eat (**C**) during 3 h after breakfast following an evening meal of RKB or WWB with or without priming (1D and 3D respectively). Values are means ± SEM. Repeated measures; mixed model in SAS. RKB, rye kernel bread; WWB, white wheat bread; 1D, single evening meal; 3D, three consecutive evening meals; VAS, Visual Analogue Scale.

The RKB evening meal increased the subjective feeling of satiety at fasting (P<0.01) and during the course of the entire experimental day (AUC 0–180 min, P<0.05), compared to the WWB evening meal (Table 6 and Fig 5). The subjective feeling of hunger and desire to eat were reduced after the RKB evening meal both at fasting (P<0.001 and P<0.01, respectively) and after the standardized breakfast (AUC 0–180 min, P<0.05), as compared to after the WWB evening meal (Table 6 and Fig 5). No significant differences between length of priming or [length of priming*product] interactions were observed.

**Table 6:** Subjective appetite ratings following one and three consecutive days of evening meals with reference- and test product, respectively^1^.

Test variables	WWB	RKB	%Δ^2^
Satiety, fasting (mm)^3^	24.1±1.9	32.6±3.0	35**
Satiety, AUC 0–180min (mm·min)^4^	6280±423	7182±351	14*
Hunger, fasting (mm)^3^	67.7±2.3	56.6±3.2	−16***
Hunger, AUC 0–180min (mm·min)^4^	11000±522	9979±407	−9*
Desire to eat, fasting (mm)^3^	75.7±2.5	66.9±3.3	−12**
Desire to eat, AUC 0–180min (mm·min)^4^	12600±509	11520±411	−9*

^1^ Data are presented as means ± SEM.

* Different from WWB P<0.05;

** P<0.01,

*** P<0.001 (ANOVA, GLM, Minitab).

^2^ The percentage change is calculated as the difference from the WWB to the RKB.

^3^ n_1D_ = 17, n_3D_ = 19.

^4^ n_1D_ = 16, n_3D_ = 18.

AUC; area under curve; RKB, rye kernel based bread including one and three days of priming; WWB, white wheat bread including one and three days of priming.

## Discussion

The purpose of the present study was to examine the metabolic effects of rye kernel based products rich in fermentable DF in a semi-acute over-night perspective with two different priming settings. The results indicate that a single evening meal with whole grain rye seems to be similarly efficient in the time perspective of 10.5–13.5 h with respect to improving the metabolic test variables determined in this study, as compared to three consecutive evenings of priming with whole grain rye. The study shows that a rye kernel based product has the potential to improve glycaemic regulation, increase gut hormones involved in metabolic- and appetite regulation, and improve perceived appetite sensations in healthy subjects in a time perspective of 10.5–13.5 hours after intake. Rye has previously shown potential to improve appetite ratings in acute postprandial studies [12–14, 25, 26] and after 3 weeks of ingestion [11] in healthy subjects, which indicates a satiating effect, but changes in gut hormones and SCFA have not been studied. Therefore, this present study is to our knowledge the first to observe simultaneous improved appetite ratings, defined as increased satiety and decreased hunger and desire to eat, combined with increased satiety hormones GLP-1 and PYY, and increased SCFA concentrations in response to rye-based products.

The present study shows that the glycaemia following the standardized breakfast post RKB evening meal was prominently reduced, in parallel to a lowering of insulin surge, indicating improved insulin sensitivity. Insulin sensitivity obtained with Matsuda index was increased by 10% after RKB compared to WWB. However, the differences did not reach statistical significance (P = 0.128). The benefits on glucose and insulin are in agreement with previous findings in healthy subjects using a similar experimental model. The findings show that evening meals consisting of barley kernel products rich in intrinsic indigestible carbohydrates decrease both the postprandial glucose response and the insulin response in the postprandial phase 0–120 min (iAUC) 10.5 to 12.5 h after intake [8]. Moreover, the lowered blood glucose increment has previously been observed in a day-long intervention study with rye kernels. It was shown that a rye kernel based breakfast lowered the cumulative day-long glucose (breakfast+lunch+dinner) iAUC compared to a WWB breakfast, i.e., up to 11.5 h after ingestion of the test product [15]. It should be mentioned that whole grain rye contains other potentially beneficial bioactive components than DF, e.g. alkylresorcinol and benzoxazinoids, which may protect against e.g. cardiovascular disease [27]. However, the H_2_ excretion at the standardized dinner, in the previously mentioned day-long study, was negatively correlated with glucose response (iAUC). It was suggested that the improved glucose regulation was mediated by mechanisms originating from gut fermentation of DF in the rye [15]. To our knowledge this is the only previous study in which metabolic test variables have been obtained after whole grain rye in a similar time perspective. In accordance with the previous study, the present study showed a significant increase of breath H_2_ concentrations, between 10.5–13.5 h after ingesting RKB as an evening meal compared to WWB, suggesting that gut fermentation may be involved in the beneficial effects seen after rye in this time perspective. Additionally, since the study design involved possible limitations in that the test subjects ingested the test products at home, not allowing control of compliance, the hydrogen measurements is considered as a complementary tool to get a quick and approximate indication of the fiber intake. The noticeable difference in breath H_2_ at the experimental day after the RKB and the WWB evening meal in our study indicate that the test subjects have followed the instructions regarding the evening meal test products.

In our study, the RKB evening meal significantly lowered the concentration of FFA at fasting, which may have contributed to the improvement seen in glucose regulation in the over-night perspective, since it has been suggested that impaired insulin signaling correlates with elevated concentrations of FFA [28]. Furthermore, it has previously been observed that there is a positive relationship between elevated propionate [7] and butyrate [9], and benefits on blood glucose regulation 10.5–13.5 h and 9.5–11.5 h, respectively, after intake of barley. In addition, the concentration of FFA was lowered in both studies [7, 9]. It has also been shown that serum FFA is reduced by rectal infusion of SCFA (acetate and propionate) [29, 30]. In this context it can be suggested that there might be a relation between lowered FFA concentrations induced by increased SCFA production after RKB, and improved glucose tolerance in the present study.

Another important finding in the present study was the observed significant increase of the gut hormone p-GLP-1 the morning after the RKB evening meal. The incretin GLP-1 has been shown to have antidiabetic effects [31, 32], associated with lowering of postprandial blood glucose concentrations [33] and improvement of insulin sensitivity [34]. Additionally, GLP-1 has been ascribed anti-obesity features [35–37] and has been shown to be inversely correlated with BMI [38]. Recently, the GLP-1 response to an oral glucose tolerance test (OGTT) was studied among individuals with normal glucose tolerance (NGT), pre-diabetes or screen-detected type 2 diabetes. It was shown that the GLP-1 response was impaired in individuals with pre-diabetes and diabetes type 2 (up to 25%) compared to NGT, and an increase of GLP-1 was positively associated with improved insulin sensitivity. Additionally, the GLP-1 response in obese and overweight individuals was lower than in normal weight individuals [39], indicating that means to induce increased levels of GLP-1 by choice of food could be important in the prevention of obesity and type 2 diabetes.

In the present study, the RKB evening meal significantly increased the levels of p-PYY 10.5–12.5 hours after intake, compared to WWB, suggesting that the RKB could improve the control of the food intake the following morning. The gut hormone PYY is secreted in response to nutrients and it has been recognized to be involved in energy homeostasis and control of the food intake [40]. Studies have shown that the concentration of meal-induced PYY are lower in obese individuals [41–43], indicating that food items which increase levels of PYY are important in the prevention of obesity. Opposite to the increase of p-PYY after RKB, there was a tendency towards a decrease in the hunger hormone ghrelin 0–180 min post breakfast after ingesting RKB evening meal and a main effect of the test products was observed (repeated measures; mixed model in SAS). This is interesting since intravenous administration of ghrelin has shown to promote food intake in healthy subjects [44].

In accordance with the beneficial effects on appetite regulating hormones in our study, the RKB evening meal increased the subjective ratings of satiety and decreased the feelings of hunger and desire to eat during the entire experimental day (AUC 0–180 min). Additionally, p-nesfatin-1 showed a tendency to increased concentrations in fasting plasma; that is 10.5 h after RKB evening meal. Nesfatin-1 has previously shown to reduce food intake and fat mass in rodents [45]. Nesfatin-1 was reported to be significantly lower at fasting in humans with T2D compared with healthy subjects [46]. Moreover, it has been shown that both BMI and blood glucose are negatively correlated to nesfatin-1 in non-obese males [47]. Further research is necessary to investigate the possible role of nesfatin-1 in prevention of obesity and T2D in humans.

In contrast to significant increases in p-GLP-1 and p-PYY after the RKB evening meal, no differences in plasma concentrations of GLP-2 were noted at the breakfast depending on the evening product. It has been shown that GLP-2 enhances formation of the epithelial barrier in obese mice, hence reducing influx of inflammatory components present in the gut lumen and counteracts systemic inflammation [48]. The results from the present study did not manage to show anti-inflammatory properties of rye as determined by s-CRP, s-IL-6, s-PAI-1 and s-IL-18. The results for inflammation are ambiguous since IL-18 showed a main effect (type of evening meal) and increased mean concentration (4%) after the RKB evening meal, whereas there was a tendency to a decrease in s-IL-6 concentrations (-19%, n.s.) the following morning after the RKB evening meal. In previous studies, investigating effects of barley evening meals, results have shown a decreased concentration of IL-6 the following morning in healthy subjects [6, 8, 19]. The IL-18 concentrations were not measured in the previously mentioned studies. It is possible that the lack of significant anti-inflammatory effect of the RKB evening meal could be due to differences in DF composition between whole grain cereals, e.g. barley and rye, or due to the differences in DF content between portions if comparing to previous studies, e.g. 15.6 g of DF in RKB compared to 20–22 g of DF in barley kernels [6, 8]. Further research is needed to fully understand cardiometabolic effects of different DF in terms of e.g. anti-inflammatory properties.

It was shown in the present study that the DF in RKB evening meal promoted an increased gut fermentation activity the next morning, indicated by increased breath H_2_ excretion and plasma SCFA concentrations. Arabinoxylans is one of the major DF components in rye; around 8.7–11.5% according to the literature [49]. In terms of colonic fermentation, it has previously been shown that the total and individual fecal SCFA (acetate, propionate and butyrate) concentrations increased after a 3 week intervention with arabinoxylans (rye flakes) compared to a western diet (in pigs) [50]. The evening meal of RKB increased the total p-SCFA and the individual main SCFA, i.e., acetate, propionate and butyrate, in the morning, independently of the length of priming used (single evening meal or three consecutive evenings prior to the standardized breakfast). However, a tendency towards increased propionate concentrations was observed after three days of priming, compared to a single evening meal (P = 0.051). This result indicates that it is possible that the concentrations of SCFA would have increased further with a longer duration of RKB intake.

As previously mentioned, the results showed that RKB induced increased release of both PYY and GLP-1. Additionally, a positive correlation between fasting levels of SCFA, acetate and propionate and mean GLP-1 concentrations was detected, suggesting a gut fermentation mediated mechanism for the beneficial effects on metabolic risk markers. The results are in accordance with previous observations in-vitro displaying SCFA induced secretion of PYY and GLP-1 from L-cells located in high density in the colonic epithelium [51]. Accordingly, SCFA were implicated as the link between colonic fermentation of inulin and oligofructose and increased release of GLP-1 and PYY in humans [52, 53]. Activation of the free fatty acids receptors (FFA)2 and FFA3 present on colonic L-cells by SCFA was put forward as a mechanism [54–56].

## Conclusions

A rye kernel based product as evening meals displayed anti-diabetic and anti-obesogenic potential in an over-night perspective as manifested by enhanced glucose regulation, increased GLP-1 and PYY the subsequent morning post a standardized breakfast. An enhanced appetite regulation in the semi-acute perspective of rye kernels was indicated by improved subjective rating scores of satiety parameters. The benefits of the rye product were accompanied by increased p-SCFA and breath H_2_, indicating a mechanism related to gut fermentation of the intrinsic indigestible carbohydrates present in this product. The correlations between SCFA and GLP-1 observed in the present work are supported by previous evidence concerning a connection between colonically derived SCFA and promotion of gut hormones important hormones in appetite- and glucose regulation (GLP-1 and PYY).

## Supporting Information

S1 CONSORT Checklist Completed CONSORT checklist. (DOCX)

S1 Protocol Trial protocol. (DOCX)
