# Supplementary material for: Rye-Based Evening Meals Favorably Affected Glucose Regulation and Appetite Variables at the Following Breakfast; A Randomized Controlled Study in Healthy Subjects
Source: PLoS One. 2016 Mar 18;11(3):e0151985. doi: 10.1371/journal.pone.0151985 (PMC4798690; doi:10.1371/journal.pone.0151985)
Supplement: S1 Protocol — (DOCX) [file pone.0151985.s002.docx]

# Protocols for the experts

***"Effects of colonic fermentation of dietary fiber on appetite regulation and metabolism"***

*English translation (using Google translator with minor necessary changes) of the Swedish trial protocol. Original Swedish protocol follows the English translation.*

## Background

The prevalence of overweight and obesity is increasing worldwide, and this trend can also be seen in young people. It is known that obesity is linked to a state of low-grade inflammation and also insulin resistance. The metabolic syndrome is a common name for a condition involving severe risk of diabetes and cardiovascular diseases and include factors such as elevated blood sugar and insulin levels, elevated blood fats, high blood pressure and abdominal obesity [1]. An increased level of chronic inflammation and elevated oxidative stress is considered to be closely linked to these states of poor health [2], [3, 4]. In 2011, the estimated number of people with diabetes were 366 million and the diagnosis for 2030 is over 550 million [5]. Nutritional prevention can be a great feature to stop this epidemic.

A connection has been demonstrated between the fermentation of indigestible carbohydrates in the gastrointestinal tract and the regulation of appetite, obesity, metabolism, and low-grade chronic inflammation [6]. Gastrointestinal hormones contribute to greater satiety and suppression of hunger. The hormone can reduce hunger by decreasing hunger seeking signals and increase satiety signals [7]. Knowledge of the relationship between the gastrointestinal microflora and its metabolism is constantly increasing and it has been suggested that the "metabolic conversation" between the gastrointestinal tract and peripheral tissues regulated by the fermentation of indigestible carbohydrates in the colon [8]. Among the signaling molecules involved in the metabolic conversation exists including glucagon-like peptides 1 and 2 (GLP-1 and GLP-2), peptide YY (PYY), gastric inhibitory hormone (GIP) and oxyntomodulin which all secreted from L cells in the small intestine and colon. The gastrointestinal hormone plays a key role in energy regulation, such as glucose and appetite regulation. Up to two thirds of the insulin that is released during a meal is due to GLP-1 and GIP stimulates its release. [9] Both GLP-1 and PYY considered antidiabetic hormone and also preventive against obesity [10]. Stimulating the release of gastrointestinal hormones via the diet is therefore a promising approach for prevention and treatment of metabolic diseases.

A carbohydrate-rich diet that results in low and steady blood glucose increase (foods with a low Glycemic Index GI) has been shown to have a positive effect in the prevention and treatment of diabetes, cardiovascular diseases and metabolic syndrome [12]. Some low-GI foods, such as whole grains, have been shown to have beneficial effects on blood glucose levels, not just acute after a meal, but also for the next meal. This so-called 'Second-meal effect "has been shown both from breakfast to lunch, [13, 14], from breakfast to dinner [15] and from a late dinner to breakfast the next day [16, 17]. The explanation for the acute hypoglycaemia after a meal is a slowed-down digestion of carbohydrates and absorption of glucose. The explanation of “second-meal effect" from breakfast to lunch have also been explained by a more prolonged digestion and absorption resulting in prolonged lowering of free fatty acids, with a concomitant increase in insulin sensitivity after taking lunch. The cause of the second-meal effect in the longer term, e.g. from breakfast to dinner or an evening meal at breakfast the next day, is not fully understood, but likely involves the mechanism behind bacterial fermentation of indigestible carbohydrates in the colon.

In previous studies, we have shown that eating a grain-based food can positively affect glucose tolerance and other risk markers of metabolic syndrome, such as IL-6, adiponectin and saturation, in a 10-12 hour perspectives [16, 17]. This improvement was correlated to increased colonic fermentation as measured by hydrogen in the breath as well as increased production of short-chain fatty acids (SCFA analyzed in plasma). Furthermore, we saw in the morning after a grain-based evening meal increased plasma concentration of the incretin hormone GLP-1; GLP-1 negatively correlated to blood glucose response after the breakfast meal. GLP-1 exerts a variety of metabolic functions, such as stimulating insulin release, increase beta cell mass, increased insulin sensitivity and inhibits glucagon. In addition to effects on glucose regulation also reduces the GLP-1 gastric emptying rate which can increase satiety and reduce energy intake [18]. Due to the positive effects on glucose regulation and saturation have GLP-1 in recent years, therefore, described as an "anti-diabetic" hormone. Data indicate that the indigestible carbohydrates either directly or indirectly via fermentation can stimulate the release of GLP-1 [19]. Colonic fermentation (hydrogen concentration in the breath as a marker) in our previous study correlated positively to saturation and negatively to the gastric emptying rate.

## Hypothesis

Increasing data suggest that there is an interaction between microbiological activity in the intestine and low-grade chronic inflammation, obesity and other metabolic disorders [6, 20]. Our hypothesis is that important prebiotic effects on metabolism, cardiovascular risk markers and appetite regulation may be obtained by the intake of cereal based foods and legumes, fruit and berries. The fermentation in the colon of opening indigestible colon substrate can have a positive effect on the metabolism, and that this effect differs depending on the choice of substrates.

## Purpose

The project for which the present application relates is to study the relationship between bacterial fermentation in the colon by indigestible substrates and systemic metabolism and satiety.

## Meaning

The current project is part of a research program (the Antidiabetic Food Centre, the AFC). The overall objective of this research is to increase knowledge in order to enable the design of foods that have a positive and preventive effect on the risk factors related to obesity, diabetes and cardiovascular diseases. The result of the project for which the application refers can contribute with increased knowledge related to the interaction between the colon fermentation and risk factors for metabolic disorders. The results of the project can also provide important information relevant to the design and development of healthy foods.

## Project description and methods

*Colon Substrates to be studied are:*

• Products that naturally contains high content of indigestible carbohydrates, such as cereals, legumes, fruits and berries.

• Food products (eg white bread) enriched with natural indigestible substrates such as dietary fiber, resistant starch and polyphenols isolated from sources containing these fermentable substrate such as cereals, legumes, fruits and berries.

*Previous experience of methods, procedures (own and others) particularly with regard to the risks and possible complications.*

In the research department where experiments performed (Division of Applied Nutrition and Food Chemistry, Lund University), we have great experience in similar studies (eg. See ref: 14, 15, 16 and 17). Blood samples are taken by a licensed nurse.

*Description of access to relevant security / staff should be described.*

Very small risk exists. The products included in the studies are common in the general diet (such as cereals, legumes or bread with added dietary fiber or berry polyphenols (the addition of concentrates of berries (such as blueberries)). Blood samples are taken by a licensed nurse.

*Ethical considerations*

If it is found that any test variables (especially blood glucose) is out of the border is considered normal informs a nurse person research on this, and provides guidance and appropriate advice. The risks of complications during the experiments are very small. The sample results are treated confidentially. All results are based and presented at the group averages. No single person can be identified in the reporting of results. Research subjects are healthy, participate voluntarily and are carefully informed that at any time can cancel the trials without giving any reason.

*Subjects*

The subjects will be healthy men and women between 20-70 years, BMI <35.

*Experimental Design*

The project is implemented as part studies:

a) Identification of the metabolic effects of the test products as the products consumed in the evening, and test parameters are measured at a subsequent standardized breakfast and if relevant even after a standardized lunch.

b) Identification of the metabolic effects of a standardized breakfast when the intake of test products are over several days (up to five days).

c) Identification of the metabolic effects of a standardized breakfast when the intake of test products are over a longer period (up to two weeks).

d) The same as a, b and c, but the probiotic bacteria are added test meals (e.g., lactobacilli and bifidobacteria (bacteria added e.g. ProViva and dairy products).

The number of test products in a sub-study is between 1-6 pieces. Furthermore, a reference product (white bread without added fiber) is used in each sub-study. Between 15 subjects and 40 subjects are included in each part of the experiment. A "cross-over" design is used, i.e. each subject tested for all test product and the reference product (a product at a time), and each person's test results for all products is compared within the same test person. The test products are administered in a randomized order at about 1 week between the two products to ensure that not there is no delayed effect of a previous product.

In total, each subject in a sub-study is not involved in trial no more than eight times (six products + a reference product on two occasions). The subjects arrive 07:30 on test day (about 3-4 volunteers / testing occasion) fasting from the night before (21:00) as a test product or reference product ingested. Test markers analyzed on samples taken fasting and repeated over 3 - 6.5 hours after a standardized breakfast served about 8:00. Samples are taken after breakfast and when it is relevant even after a standardized lunch.

*Blood tests*

Capillary blood samples for blood glucose determination fasted and then repeated (up to 15 times) over a 6.5 hour period. Postprandial blood glucose fluctuations, such as after a meal, are analyzed in capillary blood, not venous. A Venflon put into a vein in the bend of the arm and venous samples are taken at the same times as for blood glucose. Blood samples are analyzed for: blood glucose, blood lipids, insulin, intestinal hormones (GIP, GLP-1, GLP-2), FFA, SCFA, antioxidative capacity (such as SOD, catalase, glutathione peroxidase), inflammatory markers (such as IL-6, CRP, adiponectin) and satiety markers (such as Grelin, CCK, PYY, PP). The total amount of blood per trial day will be <100 mL. The total amount of blood in an attempt will be not more than 800 ml (collected over 2-4 months). Approximately times of blood tests will be at 15, 30, 45, 60, 90, 120, 150, 180, 210, 240, 255, 270, 300, 330, 360 and 390 minutes. Plasma and serum is separated by centrifugation and placed in the freezer until they are to be analyzed.

*Faecal samples*

Faecal samples will be taken to identify colon flora.

*Samples of exhaled air*

Samples of hydrogen in the breath (marker for colonic fermentation) at the same time as taking blood samples by the test subjects must exhale a deep breath in a portable hydrogen monitor. Hydrogen excretion is a measure of the fermentation in the colon.

*Feeling of Satiety*

Satiety formulary that describes the perceived subjective satiety, hunger and desire to eat may be filled in repeatedly during the 6.5 hour period.

*Energy intake*

As lunch is served, the importance of the food to be measured in order to measure the subject’s voluntary food intake.

**Protokoll för fackmän**

”*Effekter av kolonfermentering av kostfiber på aptitreglering och metabolism*”

### Bakgrund

Förekomsten av övervikt och fetma ökar i världen och denna trend kan även ses hos unga. Det är känt att övervikt är kopplat till ett tillstånd av låggradig inflammation och även till insulinresistens. Det metabola syndromet är ett samlingsnamn för ett tillstånd med allvarlig risk för diabetes och hjärt-kärlsjukdomar och inkluderar faktorer såsom förhöjda blodsocker- och insulinnivåer, förhöjda blodfetter, högt blodtryck och bukfetma [[1](#_ENREF_1)]. En ökad grad av kronisk inflammation och en förhöjd oxidativ stress anses vara starkt knutet till dessa ohälsotillstånd [[2](#_ENREF_2)], [[3](#_ENREF_3), [4](#_ENREF_4)]. År 2011 beräknades antalet personer med diabetes till 366 miljoner och diagnosen för 2030 är över 550 miljoner [[5](#_ENREF_5)]. Kostprevention kan här ha en stor funktion för att stoppa denna epidemi.

Det har påvisats samband mellan fermentering av odigererbara kolhydrater i mag-tarmkanalen och reglering av aptit, fetma, metabolism och låggradig kronisk inflammation [[6](#_ENREF_6)]. Gastrointestinala hormoner bidrar till ökad mättnadskänsla och dämpning av hunger. Hormonen kan minska hungern genom att minska hungersökande signaler och öka mättnadssignaler [[7](#_ENREF_7)]. Kunskaperna om förhållandet mellan mag-tarmkanalens mikroflora och dess metabolism ökar ständigt och det har föreslagits att det ”metabola samtalet” mellan mag-tarmkanalen och perifera vävnader regleras av fermenteringen av onedbrytbara kolhydrater i kolon [[8](#_ENREF_8)]. Bland signalmolekylerna involverade i det metabola samtalet finnes bland annat glukagonliknande peptider 1 och 2 (GLP-1 och GLP-2), peptid YY (PYY), gastriskt hämmande hormon (GIP) och oxyntomodulin vilka alla utsöndras från L-celler i tunntarmen och kolon. De gastrointestinala hormonen spelar en viktig roll i energiregleringen, som t.ex. glukos- och aptitreglering. Upp till två tredjedelar av det insulin som frisätts i samband med en måltid är på grund av GLP-1 och GIP stimulerar dess frisättning [[9](#_ENREF_9)]. Både GLP-1 och PYY anses vara antidiabetiska hormon och även preventiva mot fetma [[10](#_ENREF_10)]. Att stimulera frisättningen av gastrointestinala hormoner via dieten är därför ett lovande angreppssätt för prevention och behandling av metabola sjukdomar.

En kolhydratrik kost som resulterar i en låg och jämn blodglukosstegring (livsmedel med lågt glykemiskt index, GI) har visats ha en positiv effekt vid förebyggande och behandling av åldersdiabetes, hjärt-kärlsjukdomar och det metabola syndromet [[12](#_ENREF_12)]. Vissa låg-GI livsmedel, t ex hela kornkärnor, har visats ha gynnsamma effekter på blodsockret, inte bara akut efter en måltid, utan även efter nästkommande måltider. Denna s.k. "second-meal effect" har visats både från frukost till lunch [[13](#_ENREF_13), [14](#_ENREF_14)], från frukost till middag [[15](#_ENREF_15)] och från en sen middag till frukost dagen därpå [[16](#_ENREF_16), [17](#_ENREF_17)]. Förklaringen till den akuta blodsockersänkningen efter en måltid ligger i en förlångsammad nedbrytning av kolhydraterna och absorption av glukos. Förklaringen till "second-meal effekten" från frukost till lunch har även den förklarats av en mer utdragen matspjälkning och absorption vilket ger en förlängd sänkning av fria fettsyror, med en åtföljande höjning av insulinkänsligheten vid intag av lunchen. Orsaken till second-meal effekten i ett längre tidsperspektiv, t.ex. från frukost till middag eller från en kvällsmåltid till frukost dagen därpå, är ej helt klarlagd, men troligtvis involverar de bakomliggande mekanismerna bakteriell fermentering av odigererbara kolhydrater i tjocktarmen.

I tidigare studier har vi visat att intag av en kornbaserad måltid positivt kan påverka glukostolerans och andra riskmarkörer för metabolt syndrom, så som IL-6, adiponektin och mättnad, i ett 10-12 h perspektiv [[16](#_ENREF_16), [17](#_ENREF_17)]. Denna förbättring var korrelerad till en ökad kolonfermentering mätt med vätgas i utandningsluften samt en ökad produktion av kortkedjiga fettsyror (SCFA analyserat i plasma). Vidare såg vi på morgonen efter en kornbaserad kvällsmåltid en ökad koncentration i plasma av inkretinhormonet GLP-1; GLP-1 korrelerade negativt till blodglukosresponsen efter frukostmåltiden. GLP-1 utövar ett flertal metabola funktioner, t ex stimulerar insulinfrisättning, ökar beta-cell massan, ökar insulinkänsligheten och inhiberar glukagonfrisättningen. Förutom effekter på glukosregleringen reducerar även GLP-1 magsäckstömningshastigheten vilket kan öka mättnadskänslan och reducera energiintaget [[18](#_ENREF_18)]. På grund av de positiva effekterna på glukosreglering och mättnad har GLP-1 på senare år därför beskrivits som ett "anti-diabetiskt" hormon. Data tyder på att odigererbara kolhydrater antingen direkt eller indirekt via fermentering kan stimulera frisättningen av GLP-1 [[19](#_ENREF_19)]. Kolonfermenteringen (vätgashalten i utandningsluften som markör) i vår tidigare studie korrelerade positivt till mättnad och negativt till magsäckstömningshastigheten.

### Hypotes

Allt mer data tyder på att det föreligger ett samspel mellan mikrobiotisk aktivitet i tarmen och låggradig kronisk inflammation, fetma och andra metabola störningar [[6](#_ENREF_6), [20](#_ENREF_20)]. Vår hypotes är att viktiga prebiotiska effekter på metabolismen, kardiovaskulära riskmarkörer och aptitreglering kan fås av intag av cerealiebaserade livsmedel och legymer samt frukt och bär. Fermentering i kolon av ingående odigererbara kolonsubstrat kan ha en positiv effekt på metabolismen, och att denna effekt skiljer sig beroende av val av substrat.

### Syfte

Syftet med projektet för vilken den aktuella ansökan avser är att studera sambandet mellan bakteriefermentering i kolon av odigererbara substrat och systemisk metabolism och mättnad.

### Betydelse

Det aktuella projektet är en del i ett forskningsprogram (Antidiabetic Food Center, AFC). Den övergripande målsättningen i detta forskningsprogram är att öka kunskapen för att möjliggöra design av livsmedel som har en positiv och preventiv inverkan på riskfaktorer relaterade till fetma, åldersdiabetes och hjärt-kärlsjukdomar. Resultatet av projektet för vilken ansökan avser kan bidra med ökad kunskap relaterad till samspel mellan kolonfermentering och riskfaktorer för metabol ohälsa. Resultatet av projektet kan också ge viktig information av betydelse för design och utveckling av hälsosamma livsmedel.

### Projektbeskrivning och metoder

*Kolonsubstrat som ska studeras är:*

- Produkter som naturligt innehåller hög halt av odigererbara kolhydrater, så som cerealier, legymer, frukt och bär.
- Livsmedelsprodukter (t ex vitt bröd) som berikats med naturliga odigererbara substrat så som kostfiber, resistent stärkelse och polyfenoler, som isolerats från källor innehållande dessa fermenterbara substrat så som cerealier, legymer, frukt och bär.

*Tidigare erfarenheter av metoder, procedurer (egna och andras) särskilt med hänsyn till risker samt möjliga komplikationer.*

På forskningsavdelningen där försöken ska utföras (Avdelningen för Industriell Näringslära och Livsmedelskemi, Lunds Universitet) har vi stor erfarenhet av liknande studier (ex. se ref: 14, 15, 16 och 17). Blodprover tas av legitimerad sjuksköterska.

*Beskrivning av tillgång till relevant säkerhet/personal ska beskrivas.*

Mycket små risker förekommer. Produkterna som ingår i studierna är vanligt förekommande i den allmänna dieten (t ex cerealier, baljväxter eller bröd med tillsatts av kostfiber eller bär-polyfenoler (tillsatts av koncentrat av bär (t ex blåbär)). Blodprover tas av legitimerad sjuksköterska.

*Etiska överväganden*

Om det visar sig att någon testvariabel (framför allt blodsocker) är utanför gränsen som anses normalt informerar en sjuksköterska forskningspersonen om detta, samt ger vägledning och lämpliga råd. Riskerna för komplikationer vid försöken är mycket små. Provsvaren behandlas konfidentiellt. Alla resultat baseras och redovisas på gruppmedeltal. Ingen enskild person kan därför identifieras vid resultatredovisning. Forskningspersonerna är friska, deltar frivilligt och är noga informerade om att de när som helst kan avbryta försöken utan att ange några skäl.

*Försökspersoner*

Försökspersonerna ska vara friska män och kvinnor mellan 20-70 år, BMI < 35.

*Försöksdesign*

Projektet genomförs som delstudier:

a) kartläggning av metabola effekter av testprodukter då produkterna intas på kvällen och testparametrar mäts vid en efterföljande standardiserad frukost och om relevant även efter en standardiserad lunch.

b) kartläggning av metabola effekter vid en standardiserad frukost då testprodukterna intagits under flera dagar (upp till fem dagar).

c) kartläggning av metabola effekter vid en standardiserad frukost då testprodukterna intagits under en längre tid (upp till två veckor).

d) på samma sätt som a, b och c men probiotiska bakterier tillsätts testmåltiderna (t ex lactobaciller och bifidobakterier (bakterier som tillsätts t ex ProViva och mejeriprodukter).

Antalet testprodukter i en delstudie är mellan 1-6 st. Dessutom ingår en referensprodukt (vitt bröd utan tillsatt fiber) i varje delstudie. I varje delförsök ingår mellan 15 försökspersoner och 40 försökspersoner. En "cross-over" design används, dvs. varje försöksperson testas efter samtliga testprodukter och referensprodukt (en produkt i taget), och varje testpersons resultat efter samtliga produkter jämförs inom samma testperson. Testprodukterna intas i en randomiserad ordning med ca 1 v mellan två produkter för att säkerställa att inte det föreligger någon kvardröjd effekt av en tidigare produkt.

Sammanlagt i en delstudie deltar varje testperson i försök vid högst åtta tillfällen (sex produkter + en referensprodukt vid två tillfällen). Försökspersonerna anländer 07.30 på försöksdagen (ca 3-4 försökspersoner/testtillfälle) fastande från kvällen innan (21.00) då en testprodukt eller referensprodukt intagits. Testmarkörer analyseras på prover som tas fastande och upprepat under 3 – 6,5 timmar efter en standardiserad frukost som serveras ca 8.00. Prover tas efter frukost och när det är relevant även efter en standardiserad lunch.

*Blodprover*

Kapillärt blodprov tas för blodglukosbestämning fastande och sedan upprepat (upp till 15 gånger) under en 6,5-timmarsperiod. Postprandiella blodglukossvängningar, så som efter en måltid, analyseras med fördel på kapillärt blod, ej på venöst. En venflon sätts i en ven i armvecket och venösa prover tas genom denna vid samma tidpunkter som för blodglukos. Blodprover analyseras med avseende på: blodglukos, blodlipider, insulin, tarmhormoner (GIP, GLP-1, GLP-2), FFA, SCFA, antioxidativ kapacitet (så som SOD, katalas, glutationperoxidas), inflammations markörer (så som IL-6, CRP, adiponektin) och mättnadsmarkörer (så som Grelin, CCK, PYY, PP). Den sammanlagda mängden blod per försöksdag blir < 100 ml. Den sammanlagda mängden blod under ett försök blir högst 800 ml (samlat under 2 – 4 månader). Cirkatider för blodprover kommer att vara vid 15, 30, 45, 60, 90, 120, 150, 180, 210, 240, 255, 270, 300, 330, 360 och 390 minuter. Plasma och serum avskiljs efter centrifugering och placeras i frys tills det att de ska analyseras.

*Faecesprover*

Faecesprover kommer att tas för att kartlägga kolonflora.

*Prover på utandningsluft*

Prover på vätgas i utandningsluften (markör för kolonfermentering) tas vid samma tidpunkter som blodprovstagningen genom att försökspersonerna får andas ut ett djupt andetag i en portabel vätgasmonitor. Vätgasutsöndringen är ett mått på fermentering i tjocktarmen.

*Mättnadskänsla*

Mättnadsformulär som beskriver upplevd subjektiv mättnad, hunger och viljan att äta får fyllas i upprepat under 6,5 h perioden.

*Energiintag*

Då lunch serveras kommer vikten av maten att mätas för att kunna mäta förpersonens frivilliga matintag.

### Referenser

1. Valle, M., et al., *Low-grade systemic inflammation, hypoadiponectinemia and a high concentration of leptin are present in very young obese children, and correlate with metabolic syndrome.* Diabetes & Metabolism, 2005. **31**(1): p. 55-62.

2. Van Guilder, G.P., et al., *Influence of metabolic syndrome on biomarkers of oxidative stress and inflammation in obese adults.* Obesity (Silver Spring), 2006. **14**(12): p. 2127-31.

3. Paoletti, R., et al., *Metabolic syndrome, inflammation and atherosclerosis.* Vasc Health Risk Manag, 2006. **2**(2): p. 145-52.

4. Esposito, K., et al., *Inflammatory cytokine concentrations are acutely increased by hyperglycemia in humans: role of oxidative stress.* Circulation, 2002. **106**(16): p. 2067-72.

5. Duarte, A.I., et al., *Crosstalk between diabetes and brain: Glucagon-like peptide-1 mimetics as a promising therapy against neurodegeneration.* Biochimica et Biophysica Acta (BBA) - Molecular Basis of Disease, 2013. **1832**(4): p. 527-541.

6. Cani, P.D. and N.M. Delzenne, *The role of the gut microbiota in energy metabolism and metabolic disease.* Curr Pharm Des, 2009. **15**(13): p. 1546-58.

7. Sam, A.H., et al., *The role of the gut/brain axis in modulating food intake.* Neuropharmacology, 2012. **63**(1): p. 46-56.

8. Denise Robertson, M., *Metabolic cross talk between the colon and the periphery: implications for insulin sensitivity.* Proceedings of the Nutrition Society, 2007. **66**(03): p. 351-361.

9. Kazakos, K., *Incretin effect: GLP-1, GIP, DPP4.* Diabetes Research and Clinical Practice, 2011. **93, Supplement 1**(0): p. S32-S36.

10. Shen, J., M.S. Obin, and L. Zhao, *The gut microbiota, obesity and insulin resistance.* Molecular Aspects of Medicine, 2013. **34**(1): p. 39-58.

11. Chew, G.T., S.K. Gan, and G.F. Watts, *Revisiting the metabolic syndrome.* Med J Aust, 2006. **185**(8): p. 445-9.

12. McKeown, N.M., et al., *Carbohydrate nutrition, insulin resistance, and the prevalence of the metabolic syndrome in the Framingham Offspring Cohort.* Diabetes Care, 2004. **27**(2): p. 538-46.

13. Jenkins, D.J., et al., *Slow release dietary carbohydrate improves second meal tolerance.* American Journal of Clinical Nutrition, 1982. **35**(6): p. 1339-46.

14. Liljeberg, H.G., A.K. Åkerberg, and I.M. Bjorck, *Effect of the glycemic index and content of indigestible carbohydrates of cereal-based breakfast meals on glucose tolerance at lunch in healthy subjects.* American Journal of Clinical Nutrition, 1999. **69**(4): p. 647-55.

15. Nilsson, A.C., et al., *Effect of cereal test breakfasts differing in glycemic index and content of indigestible carbohydrates on daylong glucose tolerance in healthy subjects.* Am J Clin Nutr, 2008. **87**(3): p. 645-54.

16. Nilsson, A., et al., *Effects of GI and content of indigestible carbohydrates of cereal-based evening meals on glucose tolerance at a subsequent standardised breakfast.* Eur J Clin Nutr, 2006. **60**(9): p. 1092-9.

17. Nilsson, A.C., et al., *Including indigestible carbohydrates in the evening meal of healthy subjects improves glucose tolerance, lowers inflammatory markers, and increases satiety after a subsequent standardized breakfast.* J Nutr, 2008. **138**(4): p. 732-9.

18. Holst, J.J., *The physiology of glucagon-like peptide 1.* Physiol Rev, 2007. **87**(4): p. 1409-39.

19. Delzenne, N.M., P.D. Cani, and A.M. Neyrinck, *Modulation of glucagon-like peptide 1 and energy metabolism by inulin and oligofructose: experimental data.* J Nutr, 2007. **137**(11 Suppl): p. 2547S-2551S.

20. Cani, P.D. and N.M. Delzenne, *Interplay between obesity and associated metabolic disorders: new insights into the gut microbiota.* Curr Opin Pharmacol, 2009. **9**(6): p. 737-43.
